# New Bio-Indicators for Long Term Natural Attenuation of Monoaromatic Compounds in Deep Terrestrial Aquifers

**DOI:** 10.3389/fmicb.2016.00122

**Published:** 2016-02-09

**Authors:** Thomas Aüllo, Sabrina Berlendis, Jean-François Lascourrèges, Daniel Dessort, Dominique Duclerc, Stéphanie Saint-Laurent, Blandine Schraauwers, Johan Mas, Delphine Patriarche, Cécile Boesinger, Michel Magot, Anthony Ranchou-Peyruse

**Affiliations:** ^1^Université de Pau et des Pays de l’Adour, Institut des Sciences Analytiques et de Physico-Chimie Pour l’Environnement et les Matériaux UMR 5254, Equipe Environnement et MicrobiologiePau, France; ^2^APESAPau, France; ^3^TOTAL – Centre-Scientifique-Technique-Jean-FegerPau, France; ^4^STORENGY – Geosciences DepartmentBois-Colombes, France; ^5^TIGF – Transport et Infrastructures Gaz FrancePau, France

**Keywords:** natural attenuation, deep aquifer, BTEX, sulfate-reduction, *Desulfotomaculum*

## Abstract

Deep subsurface aquifers despite difficult access, represent important water resources and, at the same time, are key locations for subsurface engineering activities for the oil and gas industries, geothermal energy, and CO_2_ or energy storage. Formation water originating from a 760 m-deep geological gas storage aquifer was sampled and microcosms were set up to test the biodegradation potential of BTEX by indigenous microorganisms. The microbial community diversity was studied using molecular approaches based on 16S rRNA genes. After a long incubation period, with several subcultures, a sulfate-reducing consortium composed of only two *Desulfotomaculum* populations was observed able to degrade benzene, toluene, and ethylbenzene, extending the number of hydrocarbonoclastic–related species among the *Desulfotomaculum* genus. Furthermore, we were able to couple specific carbon and hydrogen isotopic fractionation during benzene removal and the results obtained by dual compound specific isotope analysis (𝜀_C_ = -2.4‰ ± 0.3‰; 𝜀_H_ = -57‰ ± 0.98‰; AKIE_C_: 1.0146 ± 0.0009, and AKIE_H_: 1.5184 ± 0.0283) were close to those obtained previously in sulfate-reducing conditions: this finding could confirm the existence of a common enzymatic reaction involving sulfate-reducers to activate benzene anaerobically. Although we cannot assign the role of each population of *Desulfotomaculum* in the mono-aromatic hydrocarbon degradation, this study suggests an important role of the genus *Desulfotomaculum* as potential biodegrader among indigenous populations in subsurface habitats. This community represents the simplest model of benzene-degrading anaerobes originating from the deepest subterranean settings ever described. As *Desulfotomaculum* species are often encountered in subsurface environments, this study provides some interesting results for assessing the natural response of these specific hydrologic systems in response to BTEX contamination during remediation projects.

## Introduction

Deep subterranean ecosystems have been described during the last decades as a key living earth component for global carbon cycling and geo-engineering system (Pedersen, 2000; Griebler et al., 2014; Wilkins et al., 2014). This is explained by the unexpected microbial biomass discovered leading to the presumption that these ecosystems potentially host an estimated biomass equivalent to about 40–60% of the terrestrial surface biomass (Whitman et al., 1998; McMahon and Parnell, 2014). However, the available ecological data concerning subterranean environments remain limited in literature, mainly because of the difficulties in collecting representative samples, especially in deep confined aquifers. Here, we imply deep confined aquifers to be geological formations located 100s of meters deep and isolated from surface interaction by an impermeable geological layer. These geological formations are often associated with active petroleum reservoirs, or depleted oil fields used for underground gas storage.

The biodegradation of hydrocarbons has long been regarded as a strictly aerobic process, depending on oxygen availability and the presence of oxygen-respiring bacteria. But during the last few decades, anaerobic hydrocarbon degradation has been described in anaerobic surface or shallow subsurface environments (Barker et al., 1987; Ball and Reinhard, 1996; Head et al., 2003, 2010; Widdel et al., 2010) by numerous consortia and several original bacterial strains (Heider et al., 1999; Widdel and Rabus, 2001; Weelink et al., 2010; Kuppardt et al., 2014). It has been shown that BTEX (Benzene, Toluene, Ethylbenzene, and Xylenes isomers) could be anaerobically degraded using a variety of terminal electron acceptors such as sulfate, nitrate, ferric iron, and CO_2_ (Anderson and Lovley, 2000; Chakraborty and Coates, 2004; Weelink et al., 2010; Holmes et al., 2011; Vogt et al., 2011). However, benzene and ethylbenzene are the most recalcitrant of these hydrocarbons. No pure strain able to degrade benzene in sulfate-reducing conditions has been isolated, and only one regarding ethylbenzene (Kniemeyer et al., 2003).

Oil biodegradation in petroleum reservoirs, and by extension in all deep subsurface environments, is an anaerobic process (Magot et al., 2000; Head et al., 2003; Widdel et al., 2010). Very little is known about bacterial species involved in similar processes in the terrestrial subsurfaces, despite the importance of oil biodegradation to the oil industry. Some studies have reported direct or indirect evidences of oil biodegradation by molecular ecology studies (Nazina et al., 2006), thermodynamic calculations (Dolfing et al., 2008; Onstott et al., 2010), isotopic fractionation and ^13^C tracer-labeled experiments in microcosms (Mancini et al., 2003; Elsner et al., 2005; Fischer et al., 2008; Jones et al., 2008; Imfeld et al., 2014) and by cultural approaches under high temperature and pressure mimicking petroleum reservoir conditions (Mayumi et al., 2011; for a review, see Head et al., 2010).

Studying the microbial ecology and microbial activities of the deep subsurface is difficult, mainly because representative samples are technically very challenging to collect in these deep environments and microbiological studies are scarce (Greksák et al., 1990; Ivanova et al., 2007; Balk et al., 2008; Balk et al., 2010). The opportunity to collect representative fluids from the deep subsurface occurred for a few years with a specific sampling protocol set up for control wells of natural gas storage in deep aquifers (Basso et al., 2005, 2009). This led to the recent observation that original anaerobic microbial communities collected from a 830 m deep gas storage aquifer were involved in the natural attenuation of BTEX in these hydrocarbon-impacted environments (Berlendis et al., 2010). Different microbial consortia were obtained depending on the culture conditions and hydrocarbons used as carbon sources. *Proteobacteria, Firmicutes* related to the *Desulfotomaculum, Chlorobi, Thermotogales, Bacteroidetes, Synergistes*, and Euryarchaeota were shown to be present in the BTEX-degrading consortia by 16S rRNA gene studies, but specific hydrocarbon degradation activity has not been linked to any specific bacterial or archaeal group. Microbial inventory investigations of potential biodegraders isolated from deep confined subterranean environments are still of crucial interest for geo-engineering activities from oil production to bioremediation strategies. Therefore, we investigated the selection of potential biodegraders from another deep gas-storage subterranean aquifer. The microbial community strongly differs from that previously reported (Berlendis et al., 2010) and was able to anaerobically degrade benzene, ethylbenzene, and toluene during 10 years of culturing. It confirms the presence of spore-forming bacteria belonging to the genus *Desulfotomaculum* as another set of autochthonous mono-aromatic hydrocarbons biodegraders in deep subsurface environments under sulfate-reducing conditions.

## Materials and Methods

### Samples

Formation water was sampled from a deep subterranean aquifer (Parisian basin, France) located at 760 m of depth in a Jurassic superior geological formation (Lusitanien, calcareous oolites). This aquifer is confined in a poorly carbonated sandstone formation by an overlaying impermeable geological layer. The aquifer is used for geological storage of natural gas. *In situ* temperature and pH were 37°C and 8.2, respectively. The total salinity of water was 1.6 g.L^-1^. Formation water and concentrated biomass (Sterivex Filter units, EMD Millipore) were collected anoxically from the wellhead of a peripheral monitoring well after a specific cleaning procedure as previously described (Basso et al., 2005). The samples filtered on-site used in this study were transported to the laboratory under anoxic conditions (GasPak^TM^ EZ, BD), stored at 4°C to avoid microbial growth and processed the day after.

### Microcosm Experiments

The concentrated microflora collected on site on 78 0.2-μm-pore-size Sterivex^TM^ filters (Millipore) were resuspended in 2.6 L of anoxic formation water. The 102-fold concentrated bacterial suspension was used as inoculum in several flasks with formation water supplemented with 0.5 g.L^-1^ NH_4_Cl, 0.3 g.L^-1^ K_2_HPO_4_, 0.3 g.L^-1^ KH_2_PO_4_, and 2 g.L^-1^ Na_2_SO_4_ for sulfate-reducing conditions, or 0.085 g.L^-1^ NaNO_3_ for nitrate-reducing conditions, or 0.3 g.L^-1^ FeIII-citrate for iron-reducing conditions, or flushed with CO_2_/H_2_ (20/80) for methanogenic/fermentative conditions. For each condition, the media were supplemented with 1 mL.L^-1^ of trace-elements solution and 1 mL.L^-1^ of vitamin solution from a sterile anoxic stock solution prepared under N_2_ (Pfennig et al., 1981; Eichler and Pfennig, 1986). One milliliter per liter of dithionite solution (0.2% w/v) was added to the media as a reducing agent and resazurine (1 mg.L^-1^) was used as a redox indicator. From all the initial conditions (sulfate-, nitrate-, iron-reducing, and methanogenesis/fermentation media), three microcosms were prepared including one with 5% (v/v) of 1 M HCl added in order to create abiotic control conditions. Fifty milliliters aliquots were distributed in 100-mL Wheaton serum bottles sealed with butyl rubber stoppers (Bellco Glass, Inc). Benzene, toluene, ethylbenzene, o-, m-, and p-xylenes were finally added (100 ppm final concentrations; Sigma–Aldrich). All manipulations were done in an anaerobic glove box (Getinge La Calhene, France) under an atmosphere of 95% N_2_ and 5% H_2_. Incubations were performed under static conditions at the deep aquifer *in situ* temperature of 37°C in the dark.

Subcultures in sulfate reducing conditions were prepared outside the glove box using synthetic water (0.5 g.L^-1^ NH_4_Cl, 0.1 g.L^-1^ MgCl_2_.6H_2_O, 2 g.L^-1^ Na_2_SO_4,_ 0.06 g.L^-1^ CaCl_2_,2H_2_O, 0.5 g.L^-1^ NaCl, 0.3 g.L^-1^ KH_2_PO_4,_ 0.3 g.L^-1^ K_2_HPO_4,_ 1 mL.L^-1^ of an anoxic solution of trace-elements (Eichler and Pfennig, 1986), the composition of which mimicked that of the formation water. Culture media were sterilized by autoclaving for 20 min at 120°C and immediately flushed under a stream of O_2_-free N_2_ gas and cooled to room temperature prior to the addition of sterile and anoxic solutions of 1 mL.L^-1^of vitamins, 2 g.L^-1^ FeCl_2_.4H_2_O, and 2.55 g.L^-1^ Na_2_S.H_2_O. The medium was adjusted to pH 8 and 50 mL aliquots were distributed in Wheaton serum bottles sealed with butyl rubber stoppers under a stream of O_2_-free N_2_. A 10% inoculum and BTEX (100 ppm) were finally added. Chronology of the different experiments during this study is provided in **Figure 2**.

### Analytical Procedure

Aqueous samples (0.3 mL) were collected by syringe through the stoppers, transferred to chromatographic vials and acidified (10 μL of 3 N HCl) for monitoring BTEX degradation periodically by SPME/GC/FID with an autosampler Combi Pal (CTC Analytics) coupled with a gas chromatograph 7890A (Agilent Technologies) equipped with a flame ionization detector. BTEX was absorbed in headspace vials during 10 s with a micro-extraction fiber (SPME, Supelco 75 μm carboxen-PDMS). Desorption time inside the GC injector was 100 s. Compounds were separated through an Optima Wax (Macherey–Nagel) column (30 m × 0.32 mm × 0.50 μm). Helium was used as a carrier gas with a constant flow rate of 1 mL min^-1^. Results were processed as the residual percentage of BTEX as (*Ct*/*Cc*) × 100, where *Ct* is the hydrocarbon concentration in the microcosm, and *Cc* the hydrocarbon concentration in the abiotic control microcosm. The resulting values were normalized to o-xylene as an internal standard.

### DNA Extraction, PCR and ssu rRNA Clone Libraries for Taxonomic Assignment

From the fifth to eighth subcultures, genomic DNA was extracted in duplicate from the microbial communities using the Powersoil DNA isolation kit (MoBio Laboratories, Carlsbad, CA, USA). For the construction of 16S bacterial rRNA gene libraries, targeted genes were amplified using the PCR Core Kit Plus (Roche Diagnostics) with the primer sets 8F/1492R or 8F/B926R (Lane, 1991; Weisburg et al., 1991). DNA amplicons were purified, cloned, sequenced, and analyzed as previously described (Stackebrandt and Goebel, 1994; Cole et al., 2003; Berlendis et al., 2010). From the different clone libraries, 226 clones were randomly sequenced. Phylogenetic analyses were carried out after aligning related sequences using the Muscle program (Edgar, 2004). Ambiguous regions were removed using Gblocks (Castresana, 2000) and phylogenetic trees were constructed using the maximum likelihood method implemented in the phyML program v 3.0 (Guindon and Gascuel, 2003). Reliability for internal branch was assessed using the aLRT test (Anisimova and Gascuel, 2006). The 16S rRNA gene sequences reported in this study were deposited in GeneBank database with accession No. KR061296 to KR061298. Archaeal primers tested were primer couples A9F (Vetriani et al., 1999)-U1492R, A9F/A958R (DeLong, 1992), and A109F (Grobkopf et al., 1998)-A958R. Amplification of the gene coding the α-subunit of benzylsuccinate synthase was carried out using the “semi-nested” protocol described previously for *Desulfotomaculum* sp. OX39 (Winderl et al., 2007) or with the primers set 7768F/8543R designed by von Netzer et al. (2013).

### Determination of Isotopic Fractionation of ^13^C-Benzene

For the determination of isotopic fractionation, the bacterial activity was stopped in four microcosms on the seventh subculture at different stages of ^13^C-benzene (SIGMA) biodegradation by acidification to pH 2 with 15% HCl. Flasks were conserved at -20°C until analysis.

Hydrogen isotope analyses were performed in duplicate by SPME-GC-TC-IRMS using a Hewlett Packard 6890 gas chromatograph connected to a Delta plus XL^TM^ mass spectrometer with a GC/TC interface (Finnigan MAT). The gas chromatograph was equipped with a DB-PETRO column (100 m × 0.25 mm × 0.5 μm film, J&W Scientific). Helium was used as a carrier gas with a flow rate of 1.7 mL.min^-1^ for hydrogen isotope analysis. The temperature program started at 35°C for 20 min isothermally, was increased at a rate of 2°C.min^-1^ to 315°C and maintained isothermally during 50 min. Vienna Standard Mean Ocean Water (VSMOW) was used as the standard for the detection of hydrogen isotope ratios and results were reproducible within ±2.5^0^/_00_.

Carbon isotope analyses were performed in duplicate by SPME-GC-C-IRMS using the same GC as described previously connected to a Delta plus XL^TM^ mass spectrometer with a GC/C interface (Analytical Precision). The column, carrier-gas and temperature program used were the same as for hydrogen isotope analyses. Vienna Pee Dee Belemnite (VPDB) was used as the standard for the analysis of carbon isotope ratios (Coplen et al., 2006) and results were reproducible within ±0.3^0^/_00_.

Element isotope ratios “δ^h^E” (where h is atomic number) are expressed in delta notation in per mil (‰) and will be specifically designed for hydrogen and carbon, δH and δC, respectively: δ^h^E [^0^/_00_] (δH or δC) = [R_sample_–R_standard_/R_standard_] ^∗^ 1000. R_sample_ and R_standard_ are the ^13^C/^12^C or ^2^H/^1^H ratios of the sample and of the internal standard, respectively. Enrichment factors for hydrogen and carbon were determined according to the logarithmic form of the Rayleigh equation using delta notation (‰) as previously described (Elsner et al., 2005; Fischer et al., 2008). ln (R_t_/R_0_) = (𝜀 /1000) ^∗^ ln(C_t_/C_0_) where R_t_/R_0_ = (δ^h^E_t_ + 1000)/(δ^h^E_0_ + 1000). R_t_ and C_t_ are, respectively, the isotopic composition and the concentration of the compound at a given time t; R_0_ and C_0_ the same variable at the starting point of the reaction. 𝜀 [^0^/_00_] represents an isotopic enrichment factor calculated from the slope of the plot ln (C_t_/C_0_) versus ln (R_t_/R_0_) multiplied by 1000 giving the 𝜀-value in per mil. The factor Λ_bulk_ expresses the slope of the linear regression for carbon and hydrogen discrimination: ∧_bulk_ =Δδ^2^ H_bulk_/Δδ^13^ C_bulk_. In agreement with previous studies, C_t_/C_0_, which is the residual concentration of the compound at the time t, is called *f* and *B* [%] is a parameter expressing the extent of the benzene biodegradation such as *B* [%] = (1–*f*) ^∗^100.

Enrichment factors were then corrected by considering enrichment factors specific for the reactive position (𝜀 _reactive position_). As benzene is a symmetrical molecule with potentially six reactive carbons and six hydrogen atoms (Fischer et al., 2008), the AKIE (Apparent Kinetic Isotope Effect) considering the intramolecular competition of carbon and hydrogen atoms, is calculated according the following equation (Elsner et al., 2005): AKIE = 1/(1 + z ^∗^ 𝜀 _reactive position_/1000) with *z* = 6 (number of atoms of an element in identical reactive positions).

### Microscopic Observation and Cell Counts

Throughout the study, microcosms were sampled and observed under phase contrast microscopy. Total cell counts were performed by DAPI-staining (4′,6′-diamidino-2-phenylindole, Sigma–Aldrich) with an Olympus BX60 epifluorescence microscope equipped with a monochrome camera (12 bits, QIClick) and with a mercury light source. Formation water (18 mL) was fixed on-site with 2 mL of 10% borax-buffered formaldehyde (37%, Sigma–Aldrich) and stored at 4°C. Ten milliliters of sample were stained with 0.5 mL DAPI stock solution (200 μg.mL^-1^) then filtered onto 0.2 μm pore-size black polycarbonate filters (Millipore) under vacuum. For the eighth subculture, counterstaining was done by filtering a mixture of 22 μL of culture with 1 μL of DAPI stock solution onto 0.2 μm pore-size black polycarbonate filters (Millipore) under vacuum. For each filter, 10 randomly selected fields were counted.

### Inhibition Tests

On the eighth subcultures, BTE degradation inhibition tests were carried out by monitoring the biodegradation periodically and injecting sodium molybdate, Na_2_MoO_4_ (10 mM final concentration) and sodium 2-bromoethanesulfonate or BES, BrCH_2_CH_2_SO_3_Na (2 mM final concentration) through butyl stoppers just after the beginning of the biodegradation.

## Results

### Benzene, Ethylbenzene, and Toluene Removal Kinetics Along Successive Enrichments

**FIGURE 1:**
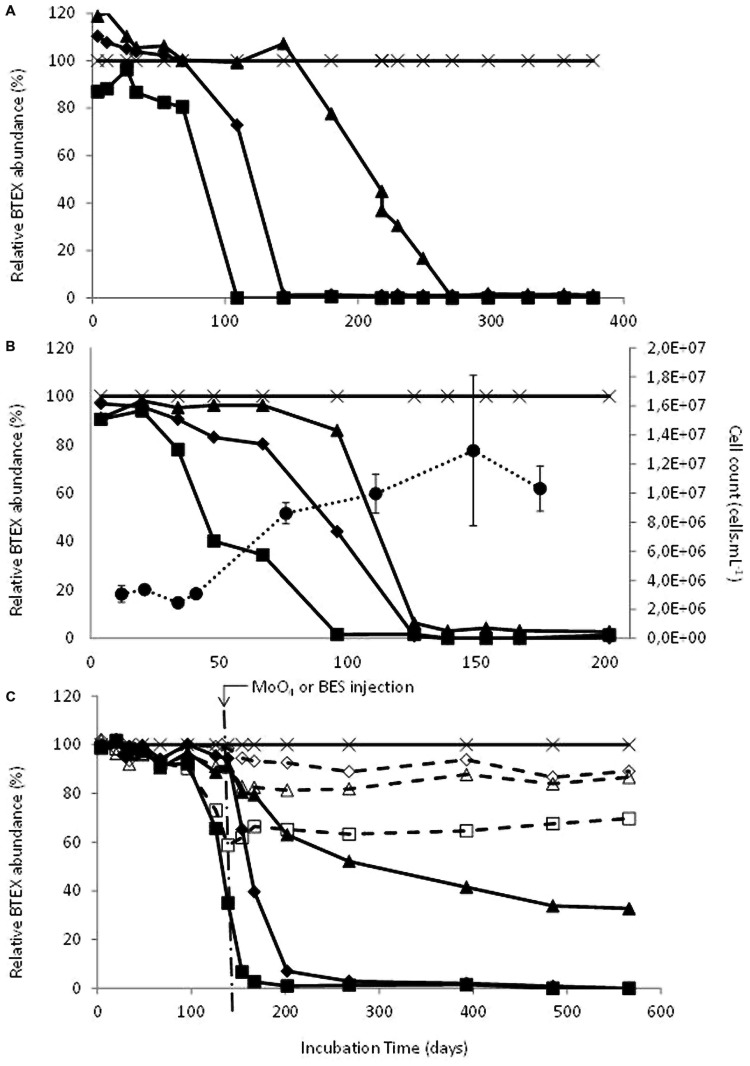
**(A)** Degradation of benzene, toluene, and ethylbenzene (BTE) during the second subculture (07/2002); *Filled squares* : ethylbenzene, *filled diamonds* : toluene, *filled triangles* : benzene, *cross* : o-xylene as internal standard. **(B)** Degradation of BTE along increase of observable biomass during the eighth subculture (04/2008); *Filled squares* : ethylbenzene, *filled diamonds* : toluene, *filled triangles* : benzene, *cross* : o-xylene as internal standard and *filled circles*: cells.mL^-1^. **(C)** Effects of inhibitors addition on BTE biodegradation during the eighth subculture (04/2008); Arrow indicates the addition at day 139 of sodium molybdate (MoO_4_, 10 mM) or bromoethanesulfonate (BES, 2 mM); *Filled squares* : ethylbenzene + BES, *filled diamonds* : toluene + BES, *filled triangles* : benzene +BES, *cross* : o-xylene as internal standard + BES or + MoO4, *open squares* : ethylbenzene + MoO_4_, *open diamonds* : toluene + MoO_4_, *open triangles* : benzene + MoO_4_. Start levels of BTEX were 100 ppm.

**FIGURE 2:**
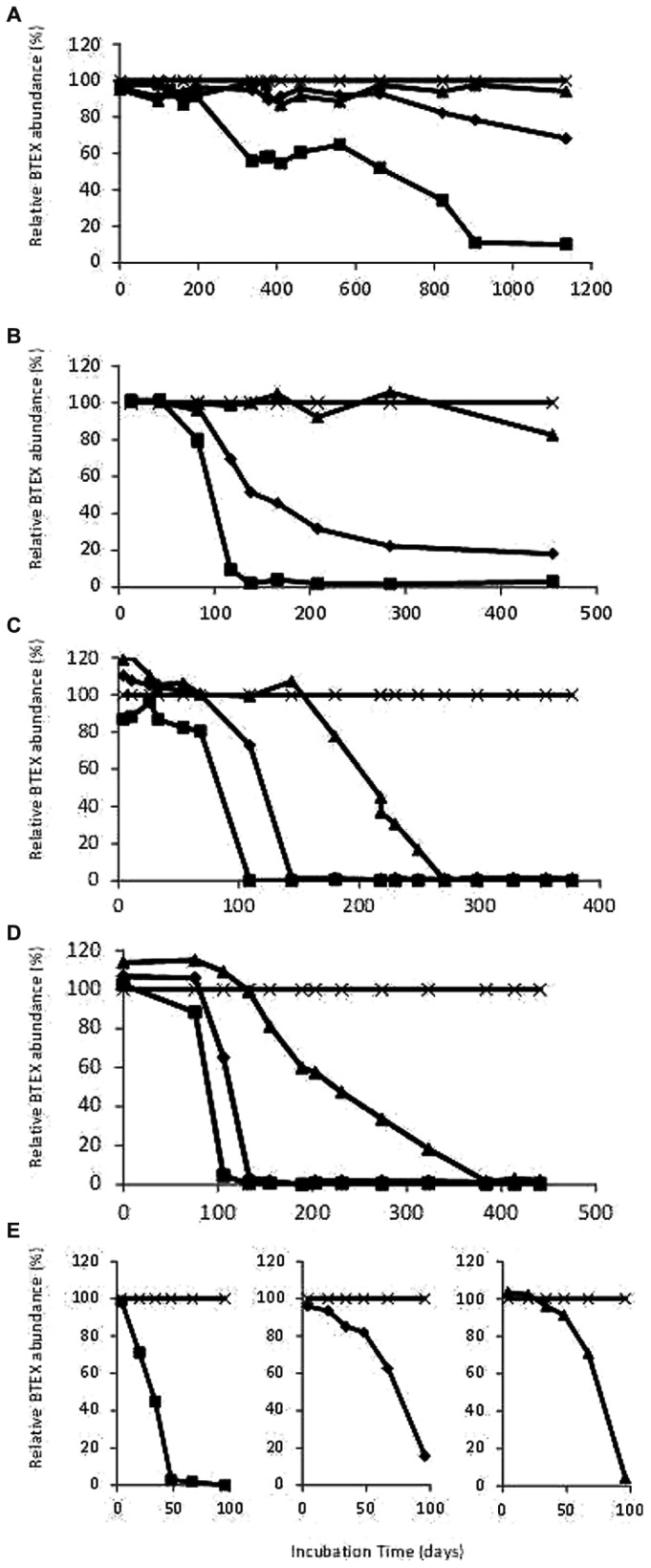
**(A)** Degradation of benzene, toluene, and ethylbenzene (BTE) during the original microcosm (10/2000) incubation; **(B)** Degradation of BTE during the first subcultures (02/2002); **(C)** Degradation of BTE during the second subcultures (07/2002); **(D)** Degradation of BTE during the fifth subcultures (01/2005). **(E)** Degradation of ethylbenzene, or toluene, or benzene during the sixth subcultures (04/2008). *Filled squares*: ethylbenzene, *filled diamonds*: toluene, *filled triangles*: benzene, *cross*: o-xylene as internal standard. Start levels of BTEX were 100 ppm.

Deep aquifer water from the original microcosm (November 2000) showed degradation of ethylbenzene after 900 days incubation under sulfate-reducing conditions. The first subculture (February 2002) was able to degrade ethylbenzene in 100 days and showed the beginning of toluene degradation whereas the second subculture (July 2002) degraded ethylbenzene in 100 days, toluene in 150 days and benzene in 270 days (**Figures 1A** and **2**). The ability to sequentially degrade ethylbenzene, toluene, and benzene (BTE) was observed for the next enrichments under sulfate-reducing conditions (from the second subcultures to the eighth ones), but no removal of xylene isomers was observed (**Figures 1** and **2**). Degradation was not observed in the abiotic controls, although there was slow mono-aromatic hydrocarbon absorption by the butyl septa as reported in similar studies (Shen and Sewell, 2005; Holmes et al., 2011). After 38 months of incubation, no BTEX removal was detected in the presence of the electron acceptors: nitrate, iron III, and CO_2_. Eight successive enrichments were achieved under sulfate-reducing conditions during the eight following years. The initial observation of the biomass in the formation water collected in November 2000 and before any transfer, showed a low bacterial biomass (8.5 × 10^3^ cells.mL^-1^) with apparent low morphological cell diversity. Subcultures of the BTE-degrading microcosms under sulfate-reducing conditions lead to a significant gain in biomass along the successive enrichments obtained years after years (**Figure 1B**). Degradation started after a lag phase lasting from 50 to 120 days and benzene degradation was complete approximately 4 months later. Whereas BES, an inhibitor of methanogenesis, addition did not show any effect, BTE degradation was significantly stopped as soon as sodium molybdate (NaMoO_4_), a specific inhibitor of sulfate reduction, was injected as shown in **Figure 1C**. It was particularly apparent with ethylbenzene where the removal process was stopped at 60% immediately molybdate was introduced. No further BTE disappearance was then observed once MoO_4_ was introduced. We also tested the BTE-degrading microbial community with ethylbenzene (100 ppm), or toluene (100 ppm), or benzene (100 ppm) as the sole carbon and energy sources. In these last assays, the toluene and the benzene removal rates were higher than in the BTEX mixture enrichment with a complete disappearance within 3 months (**Figure 2**).

### Isotopic Fractionation Associated to Anaerobic Benzene Degradation

**FIGURE 3:**
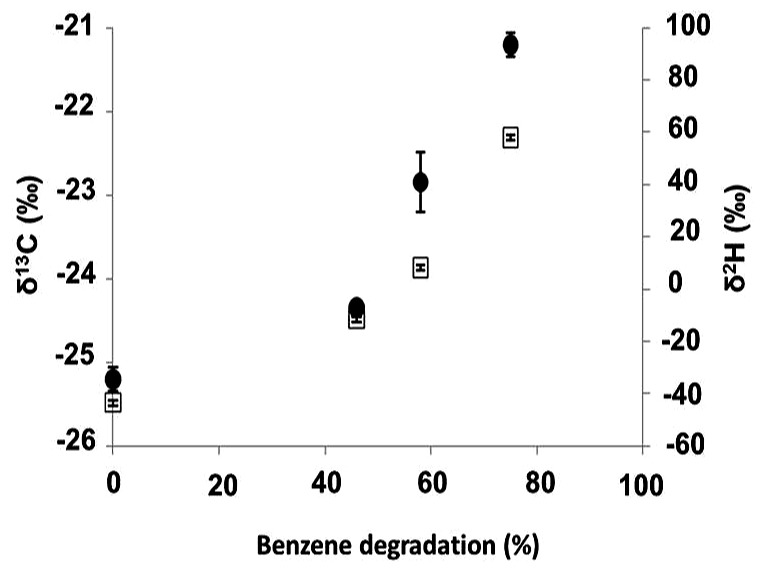
**δ^13^C and δ^2^H of residual benzene fraction versus benzene degradation rate under sulfate-reduction.** *Filled circle*: δ^13^C; *clear square*: δ^2^H. Benzene degradation refers to *B* [%], see Experimental procedures section. Start level of benzene was 12 ppm.

**FIGURE 4:**
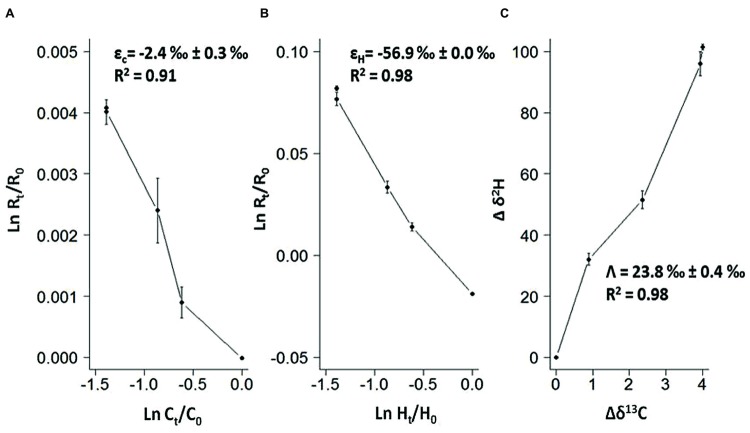
**(A,B)** Double logarithmic plot according to the Rayleigh equation expressing changes in isotopic composition and compounds concentration along time for carbon and hydrogen during anaerobic degradation of benzene; **(C)** Dual isotope plots of Δδ^2^H versus Δδ^13^C for anaerobic benzene biodegradation giving the Λ values as the slope of the regression. Dashed lines in all graphes represent the corresponding 95% confidence intervals from duplicate analysis.

**FIGURE 5:**
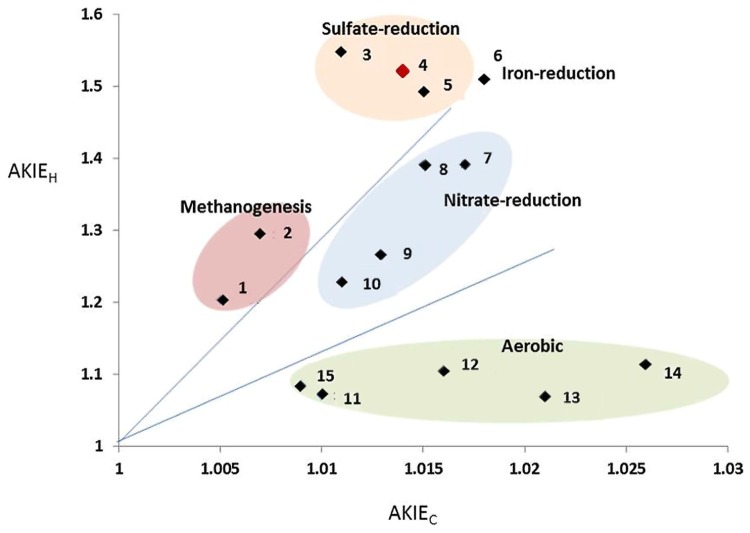
**Biplot of AKIE_C_ vs. AKIE_H_ values from data retrieved in this study and in literature dedicaced to benzene biodegradation.** Redox condition are deduced from the culture condition where the biodegradation was observed. 1: methanogenic consortium (Mancini et al., 2008); 2: methanogenic consortium (Mancini et al., 2008); 3: sulfate-reducing consortium (Fischer et al., 2008); 4: sulfate-reducing consortium, this study; 5: sulfate-reducing consortium (Bergmann et al., 2011); 6: iron-reducing consortium (Bergmann et al., 2011); 7: nitrate-reducing consortium (Mancini et al., 2003); 8: Nitrate-reducing consortium (Mancini et al., 2008); 9: nitrate-reducing consortium (Mancini et al., 2008);10: nitrate-reducing consortium (Mancini et al., 2003); 11: *Ralstonia Pickettii* (Fischer et al., 2008); 12: *Azoarcus denitriificans* (Fischer et al., 2008); 13: *Burkholderia* sp. (Hunkeler et al., 2001); 14: *Cupriavidus necator* ATCC 17697 (Fischer et al., 2008); 15: *Acinetobacter* sp. (Hunkeler et al., 2001).

Further investigation performed in the benzene-only amended microcosm showed that organic chemistry of the residual benzene was also affected along the degradation (noted *B* [%]). The initial δ^13^C and δ^2^H values of the labeled benzene were -25.2 ± 0.1‰ and -43.5 ± 0.7‰, respectively. The analysis of the residual benzene fraction revealed a significant and regular increase of δ^13^C up to -21.2 ± 0.1‰ and δ^2^H up to 58.0 ± 1.4‰ linked to the extent of benzene degradation (**Figure 3**). Sterile controls under sulfate-reducing conditions with different benzene concentrations added showed no isotopic fractionation for carbon and hydrogen, with stable carbon and hydrogen isotopes signatures over time (data not shown). The specific apparent kinetic effect of the isotopic fractionation for carbon and hydrogen (AKIE_C_ and AKIE_H_) along the benzene removal were, respectively, 1.0146 ± 0.0009 and 1.5184 ± 0.0283 and were derived from enrichment factors (𝜀_C_ and 𝜀_H_) given in **Table 1**; **Figures 4A,B** and **5**. The general combined effect of carbon and hydrogen isotopic fractionations was gaged by the ratio ∧ (∧ = 23.8 ± 0.4) obtained by the dual plot analysis of the carbon vs. hydrogen isotope fractionation range along the degradation (**Figure 4C**).

**Table 1:** Carbon and hydrogen isotope enrichment factors retrieved in this study and compared with literature data obtained during anaerobic degradation of benzene.

	Initial benzene added [μM]	𝜀C bulk [‰] ± 95 % CI [‰]	*R*^2^	𝜀H bulk [‰] ± 95 % CI [‰]	*R*^2^	Reference
**Pure culture**						
*Ralstonia picketti VKOl(omc)*	885	-1.7 ± 0.2	0.98	-11 ± 4	0.86	Fischer et al., 2008
*Cupriavidus necator* ATCC 17697 (oxic)	1180	-4.3 ± 0.4	0.99	-17 ± 11	0.89	Fischer et al., 2008
*Burkholderia* sp. (oxic)	700	-3.5 ± 0.3	0.97	-11 ± 2	0.91	Hunkeler et al., 2001
*Acinetobacter* sp. (oxic)	700	-1.5 ± 0.8	0.99	-13 ± 1	0.99	Hunkeler et al., 2001
*Azoarcus denitrificans* strain BC (oxic)	603	-2.6 ± 0.8	0.97	-16 ± 4	0.97	Fischer et al., 2008
*A. denitrifans* strain BC (Chlorate-reducing)	462	-1.5 ± 0.5	0.86	-28 ± 6	0.98	Fischer et al., 2008
**Mixed cultures**						
Nitrate-reducing, mixed negative	250	-2.2 ± 0.4	0.98	-35 ± 6	0.91	Mancini et al., 2003
Sulfate-reducing, mixed negative	192	-3.6 ± 0.3	0.92	-79 ± 4	0.79	Mancini et al., 2003
Sulfate-reducing, mixed negative	192	-1.9 ± 0.3	0.97	-59 ± 10	0.99	Fischer et al., 2008
Methanogenic, mixed negative	750	-1.9 ± 0.1	0.98	-60 ± 3	0.92	Mancini et al., 2003
Methanogenic, mixed negative	900	-0.8 ± 0.2	0.93	-34 ± 8	0.88	Mancini et al., 2008
Methanogenic, mixed negative	450	-1.1 ± 0.1	0.88	-38 ± 6	0.80	Mancini et al., 2008
Sulfate-reducing, enriched positive	450	-2.5 ± 0.2	0.97	-55 ± 4	0.93	Bergmann et al., 2011
Iron-reducing, enriched positive	200	-3.0 ± 0.5	0.93	-56 ± 8	0.93	Bergmann et al., 2011
**Sulfate-reducing, enriched positive**	**400**	**-2.4 ± 0.3**	**0.91**	**-57 ± 0.0**	**0.98**	**This study**

### Microbial Characterization of the Hydrocarbonoclastic Enrichment

**FIGURE 6:**
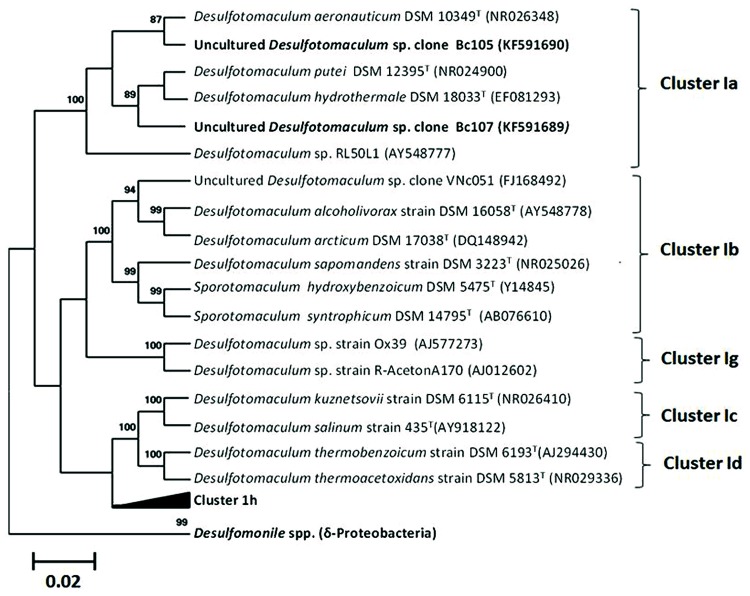
**Maximum-likelihood tree based on 16s rRNA gene (1115 bases) showing the phylogenetic relationship between the both sequences detected in microcosms (clone Bc105: minor phylotype and clone Bc107: dominant phylotype) and closest relatives.** Reliability values (aLRT values) greater than 50% are given at nodes.

Cell counts in the BTE-degrading enrichments showed that BTE removal was linked to a fourfold increase of cells in microcosms increasing from 3.6 × 10^6^ to 1.2 × 10^7^ cells.mL^-1^ (**Figure 1B**). However, the shortest doubling time about approximately 20 days was extremely low. Spores were observed from the initial enrichments (November 2000) and were still observed along the enrichments. No evidence of archaeal populations could be detected by biomolecular approaches (Archaeal 16S rRNA gene amplification). The clone libraries analyses based on 8F-926R or 8F-1492R amplicons revealed only two distinct phylotypes, both affiliated to the genus *Desulfotomaculum* (**Figure 6**). The comparison of the two related nucleic sequences based on 1492 nucleotides exhibited divergence above 5.7% between these two phylotypes. The phylotype Bc107 covered 98% of the clone libraries from the both clone libraries obtained by both primers couple, the second one, so-called, Bc105 clustered only 2% of the total clone library. Complementary microscopic observations and biomolecular approaches by t-RFLP analysis confirmed the low diversity obtained with only two distinct peaks (data not shown). The dominant phylotype Bc107 shared the closest affiliation with environmental sequences obtained from borehole water in a deep South African gold mine (clone TTMF126, accession number AY741686). The more closely related environmental sequences for the minor phylotype Bc105 were derived from a 896 m-deep aquifer linked to the South African gold mine (96% similarity with the clone DR9IPCB16SCT7, accession number AY604051) and a petroleum-contaminated soil (96% similarity with the clone EK CK572, accession number JN038217). Bc107 population shared the closest sequence similarity with thermophilic and moderate thermophilic strains in the cluster Ia of the *Desulfotomaculum* genus (Gram-positive Bacteria), such as *D. putei* isolated from the deep subterranean biosphere at 2.7 km depth (Liu et al., 1997), *D. hydrothermale* isolated from a terrestrial hot spring (Haouari et al., 2008), or *D. varum* a moderately thermophilic bacterium from a 66°C- Great Artesian Basin (Ogg and Patel, 2011).

## Discussion

### Assessment of Biodegradation Process at the Origin of the BTE-and Benzene Removal Under Sulfate-Reducing Condition

The natural gas stored in underground gas storage aquifers is mainly composed of methane but also contains traces of other compounds, which include BTEX, with concentrations in parts per billion (ppb). The majority of BTEX is withdrawn at the same time as the natural gas is extracted from the underground reservoir; however, during storage a part of the BTEX dissolves in the formation water where these compounds are undesirable. In surface environments, or in shallow aquifers with direct influences from surface environments, microorganisms are exposed to hydrocarbons naturally present in the environment (alcanes, terpenoïds) or introduced by human activities (oil spill). In the case of very deep environments (below -100 m), ecosystems have remained remarkably stable over geological time. After 150 million years isolated from the surface, gas injection with the input of a trace amount of organic matter represents an unknown stress to indigenous microorganisms which could lead to the selection of specific populations among the indigenous microbial community.

Our results showed that a deep subsurface confined pristine aquifer can hold microbial communities acting as a key player in the natural attenuation of benzene and alkylbenzenes. Our evidence is consistent with a biological, rather than a physical–chemical process, causing the disappearance of benzene, toluene and ethylbenzene (BTE): (i) repeatability over time (eight subcultures); (ii) degradation of benzene, or toluene, or ethylbenzene as sole carbon sources but no xylene degradation; (iii) an increase of biomass linked to the degradation of the monoaromatic hydrocarbons, in particular the ethylbenzene (**Figures 1** and **2**). Isotopic fractionation studies along with biological processes have been reported for decades as a promising technique for *in situ* direct evidences of biodegradations (Borden et al., 1995; Kelley et al., 1997; Ahad et al., 2000; Meckenstock et al., 2004; Zwank et al., 2004; Bergmann et al., 2011; Braeckevelt et al., 2012). Here, the extent of carbon isotope fractionation was about 4‰ which is in agreement with the extent of isotopic fractionation expected for this compound for biodegradation (>2‰); for hydrogen isotope fractionation, the change was about 100‰ which is in the same range as reported in the review by Braeckevelt et al. (2012) with values >20‰ as a proof of biodegradation process.

Other evidence, strongly suggests that sulfate-reducing microorganisms are involved in the BTE degradation. Such as the lack of degradation with electron acceptors other than sulfate (nitrate, iron(III), carbon dioxide). Archeal 16S rRNA gene was not amplified suggesting the absence of this domain in this community, in particular methanogenic archaea. This was confirmed by the inhibition of degradation with molybdate but not with BES. Additionally, the dual plot analysis of CSIA with C and H showed that our ∧ value (23.8 ± 0.4 with *R*^2^ = 0.98; **Figure 4C**) was well integrated with literature values obtained in low redox potential conditions including fermenting, methanogenic and sulfate-reducing benzene-degrading enrichments (∧ = 22–28). In contrast, all these values strongly differ with ∧ reported in literature for benzene degradation in higher redox conditions like in nitrate-reducing condition (∧ = 12–16) (Mancini et al., 2003, 2008; Fischer et al., 2007, 2008; Bergmann et al., 2011; Gieg et al., 2014). Moreover, enrichment factors obtained during anaerobic benzene degradation under sulfate-reducing conditions showed the influence of microbial composition on the extent of the isotopic fractionation of C and H among sulfate-reducing benzene degrading enrichments: our results agree with values obtained by Bergmann et al. (2011) with a Gram-positive enriched benzene degrading community (𝜀_C_ = -2.5 ± 0.2‰ with R^2^ = 0.97; 𝜀_H_ = -55 ± 4‰ with *R*^2^ = 0.93) but are different from values obtained with mixed Gram-negative sulfate-reducing enrichments (𝜀_C_ = -3.6 ± 0.3‰ with *R*^2^ = 0.92; 𝜀_H_ = -79 ± 4‰ with *R*^2^ = 0.79 [Mancini et al., 2003) and 𝜀_C_ = -1.9 ± 0.3‰ with *R*^2^ = 0.97; 𝜀_H_ = -59 ± 10‰ with *R*^2^ = 0.99 (Fischer et al., 2008)]. Our unsuccessful attempt to isolate a pure strain able to degrade at least one of the BTE compounds could be due to the existence of an obligatory syntrophism between the two detected populations at the origin of the BTE degradation as it has been previously described for the anaerobic degradation of toluene by syntrophic fermentative oxidation with a co-culture containing a sulfate-reducing bacterium (Meckenstock, 1999).

The addition of fumarate has previously been shown to be the mechanism of activation of toluene and ethylbenzene under sulfate-reducing conditions (Rabus and Heider, 1998; Kniemeyer et al., 2003). These reactions are catalyzed by fumarate-adding enzymes (FAEs) which encompass benzylsuccinate-synthase (Bss) and alkylsuccinate-synthase (Ass). PCR amplifications targeting *bssA* gene were unsuccessful using previously described specific primers (Winderl et al., 2007; von Netzer et al., 2013). The absence of known metabolic genes has also been reported in other BTEX degrading consortia dominated by Gram-positive bacteria (Hermann et al., 2008; Abu Laban et al., 2009; Bergmann et al., 2011). For two axenic BTEX degrading nitrate-reducing strains: *Dechloromonas aromatica* RCB and *Dechloromonas* sp. JJ (Coates et al., 2001; Chakraborty et al., 2005), functional genes research investigations and genomic analyses did not highlight any known pathways for anaerobic degradation of aromatics such as the central benzoyl-CoA pathway for monoaromatics and benzylsuccinate synthase (bssABC) genes for toluene and m-xylene degradation (Salinero et al., 2009). In our study, the absence of benzylsuccinate-synthase activity remains unclear. It can be postulated that if present in our community, this enzyme and its genes are different enough from those previously described to prevent gene amplification by known primers. Similar difficulties have already been reported for the *bssA* gene in toluene-degrading communities (Sun et al., 2013) and with the toluene-degrading strain *Desulfotomaculum* sp. Ox39 (Winderl et al., 2007). Alternatively, the isotopic signature of carbon and hydrogen obtained both here and previously by Bergmann et al. (2011) could be related to an unknown pathway for the anaerobic biodegradation of monoaromatic compounds and possibly specifically to a lineage among sulfate-reducing Gram-positive biodegraders. Derived values from isotopic fractionation of carbon and hydrogen, especially AKIE_C_ and AKIE_H_, published here in **Figure 5** (1.0146 ± 0.0009 and 1.5184 ± 0.0283, respectively) were perfectly integrated with other AKIE indices available in literature and showed a specific clustering among other benzene-degrading conditions with sulfate-respiring cultures. Our data will reinforce the limited existing research in term of anaerobic biodegradation of benzene available in the literature (Mancini et al., 2003; Fischer et al., 2008; Bergmann et al., 2011). Dual plot carbon specific isotope analysis, especially AKIE indices, initially suggested by Elsner et al. (2005) and reviewed by Braeckevelt et al. (2012) enables a wider range of comparisons across the biodegradation kinetics of different hydrocarbons and remains one of the more promising monitoring techniques which may, in the long term, discriminate the biochemical pathway and energy sources involved during *in situ* natural attenuation in subterranean environments.

### A New Hydrocarbonoclastic Microbial Population Representative of the Deep Subsurface Confined Aquifer

The regular observation of spores along the successive BTE-degrading enrichments and benzene-degrading enrichments strongly suggest that spore-forming microorganisms play an important role in the BTE degradation. Molecular biology approaches revealed the presence of only two different species and both belonging to *Desulfotomaculum* genus (*Firmicutes/Clostridia/Clostridiales/Peptococcaceae*, Widdel, 2006). *Desulfotomaculum* sp. and related Gram-positive sulfate-reducing bacteria such as *Desulfosporosinus* sp. are frequently encountered in deep environments (Daumas et al., 1988; Nilsen et al., 1996; Ehinger et al., 2009; Aüllo et al., 2013) and are sometimes the main representatives of these communities (Baker et al., 2003; Moser et al., 2003; Detmers et al., 2004; Moser et al., 2005), especially in several oilfields over the world with generally a positive correlation of cell abundance with increasing temperature with depth (Aüllo et al., 2013; Guan et al., 2013). Their presence in these environments could be linked to their ability to sporulate allowing them to withstand adverse periods during burial such as lack of nutrients or heat (O’Sullivan et al., 2015), and their metabolic versatility as demonstrated by their ability to respire sulfate, thiosulfate, sulfur, sulfite, metals, and metalloids. Available data on microbial diversity in deep aquifers used as natural gas storage are scarce. Nevertheless, others studies on other deep aquifers revealed the presence of this genus sometimes abundantly (Basso et al., 2009; Ehinger et al., 2009; Berlendis et al., 2010). Although members of *Desulfotomaculum* can also be found in surface ecosystems, both populations identified in this study are close to environmental sequences detected in deep subsurface environments supporting their ecological relevance (Moser et al., 2005; Gihring et al., 2006; Baito et al., 2015) (AY604051, AY741686, AB910321). On the basis of their sequences encoding the 16S rRNA gene, we assume that we are dealing with two different species. Some members of *Desulfotomaculum* and *Desulfosporosinus* have the ability to degrade hydrocarbons, in particular mono-aromatic hydrocarbons such as toluene, m-xylene and o-xylene (Robertson et al., 2000; Liu et al., 2004; Morasch et al., 2004; Abu Laban et al., 2015). Until now, bacterial isolates have shown the ability to degrade benzene either in iron-reduction (Holmes et al., 2011; Zhang et al., 2012) or in nitrate-reduction (Coates et al., 2001; Chakraborty et al., 2005; Kasai et al., 2007). Here, we demonstrate that some members of *Desulfotomaculum* are also able to degrade benzene and ethylbenzene under sulfate-reducing conditions. Despite multiple assays, no pure isolates able to degrade benzene were obtained confirming the difficulty to obtain a pure sulfate-reducing strain able to degrade anaerobically benzene (Vogt et al., 2011; van der Zaan et al., 2012), which requires us to hypothesize a possible synergy between these two populations of *Desulfotomaculum*. However, why have these organisms the capacity to degrade BTE in a poorly carbonated sandstone deep aquifer? What we know about this type of aquifers implies that the indigenous microbial communities before gas storage had to be essentially based on using CO_2_ and H_2_ on the principle of a subsurface lithoautoautrophic microbial ecosystem (SLiME) as mentioned by Stevens and McKinley (1995) and Basso et al. (2009). Having a hydrocarbon biodegradation ability could provide a benefit to *Desulfotomaculum* which can switch to various energy sources along with post-diagenetic environmental changes and consume this type of molecule potentially present as residues in a fossil organic matter entrapped in rocks. Alternatively, these microorganisms may have the same origin as the injected gas and would therefore be derived from an oil reservoir. This hypothesis would explain the ability of these organisms to degrade hydrocarbons, in particular BTEX. The natural gas treatment process after extraction from the oil reservoir, and especially the dehydration steps would allow only few spores to resist. Recently, it has been shown that *Desulfotomaculum* spores could resist triple autoclaving processes (O’Sullivan et al., 2015). Spores could then be transported thousands of kilometers in pipelines and co-injected with natural gas into deep aquifers. A previous report of the isolation of *Desufotomaculum thermocisternum* from the North sea oil reservoir able to grow syntrophically with a methanogen (Nilsen et al., 1996), the presence of *Desulfotomaculum* and methanogen species dominating the microbial diversity of a deep gold mine at 4–6 km of depth (Moser et al., 2005) support the hypothesis of a versatile metabolism in various subsurface habitats.

Many studies suggest a possible key role of Gram-positive of members of *Clostridia* (Winderl et al., 2010) and the family *Peptococcaceae* in BTEX biodegradation (Phelps et al., 1998; Da Silva and Alvarez, 2007; Kunapuli et al., 2007; Kleinsteuber et al., 2008; Musat and Widdel, 2008; Oka et al., 2008; Abu Laban et al., 2009; Berlendis et al., 2010; Taubert et al., 2012; van der Zaan et al., 2012; Kuppardt et al., 2014). This study demonstrates the key role of sulfate-reduction in this community (**Figure 1C**) and we could deduce that *Desulfotomaculum* from the community (Bc105 or Bc107 or both) degrade the aromatic hydrocarbons by direct oxidation. This BTE degradation would be similar to those described by Abu Laban et al. (2009). These authors clustered the highly abundant sequences between the genera *Desulfotomaculum* and *Pelotomaculum* and postulated these organisms were key players in benzene degradation with sulfate as the electron acceptor. The slow kinetic removal of monoaromatic hydrocarbons, and benzene in particular, observed in our study compared to kinetics reported in literature (Oka et al., 2008; Abu Laban et al., 2009; van der Zaan et al., 2012) could be linked to (i) an inhibition of anaerobic benzene degradation by co-contaminants (Edwards and Grbić-Galić, 1992; Cunningham et al., 2001; Ruiz-Aguilar et al., 2003; Da Silva and Alvarez, 2007; Vogt et al., 2011); (ii) microbial metabolism under extreme energy limitation (Hoehler and Jørgensen, 2013) and (iii) syntrophic consortia requiring optimal conditions (Vogt et al., 2011). Syntrophism between a fermentative and a by-products utilizer could be a logical adaptive strategy of indigenous lithoautotrophic communities to the presence of recalcitrant hydrocarbons. This is supported by a previous investigation using DNA-SIP techniques from a sulfate-reducing community degrading benzene enriched from contaminated groundwater, showed the likely dominance of still undescribed Gram-positive species seemingly active at the early stage of the benzene degradation followed by an undescribed Epsilon-proteobacteria assumed to be a hydrogen scavenger (Hermann et al., 2008). In the same way, a study by protein-SIP applied on a benzene-degrading microbial consortium from a shallow aquifer, Taubert et al. (2012) hypothesized the key role of *Clostridiales*, in particular *Desulfotomaculum* and *Pelotomaculum* genera, for benzene degradation. These authors and others (Kleinsteuber et al., 2008) suggested *Peptococcaceae* could putatively ferment benzene and excrete by-products such as acetate and hydrogen which would be used by the whole community, in particular Delta-proteobacteria (sulfate-reducers). If this hypothesis is true, the inhibition of sulfate-respiration after adding molybdate could stop the benzene degradation since the reaction would be thermodynamically unfavorable.

### New Bio-Indicator Parameters (AKIE Values, Phylotypes) for Benzene Biodegradation: Field Applicability in Deep Subterranean Environments

The selection of an active benzene and alkylbenzenes-degrading community shows that these hydrocarbonoclastic populations could be persistent and certainly able to sustain *in situ* biodegradation as long as a substrate is available. Deep subsurface confined aquifers despite restricted access remain key location points for subsurface engineering activity (water resources, oil, and gas industries, geothermal energy, bioremediation, fundamental research interests). However, field biodegradation studies are currently mandatory for bioremediation, oil recovery or geological exploration. Indeed, in the context of hydrocarbon biodegradation strongly linked to aquifers properties (Warren et al., 2004), petroleum industries, bioremediation experts, and environmental ecologists point out the need to systematically collect and share various technical information requiring a strong fundamental research background such as microbial ecology, isotopic studies, and biogeochemistry (Scow and Hicks, 2005; Declercq et al., 2012; Hubbard et al., 2014). Because of the higher benzene persistence in anoxic environments than its others alkylated derivatives, we focused our study on this hydrocarbon. Sequences of both phylotypes showed no close affiliation with any *Desulfotomaculum* species detected in biphenyl or benzene-degrading consortia reported in literature (Abu Laban et al., 2009; Selesi and Meckenstock, 2009). In addition, both phylotypes were loosely affiliated to any known hydrocarbonoclastic microorganisms, such as *Desulfotomaculum* sp. Ox39 (Morasch et al., 2004). Hence the results presented here obtained with this new benzene-degrading enrichment significantly extent the diversity of Gram-positive biodegraders from a deep subterranean aquifer (Abu Laban et al., 2009; Bergmann et al., 2011: for the last review about microorganisms involved in anaerobic biodegradation of petroleum, see Widdel et al., 2010). As microbial diversity in low-energy environments is known to contain only a few cultivated microorganisms, with their biochemistry and physiology largely unknown (Hoehler and Jørgensen, 2013), the additional information provided by DNA-based microbial identification such as AKIE may enable further studies with similar results to bring new evidences and new insights about these biodegradation processes. For example, a conceptual model of syntrophic biodegradation of hydrocarbons has been initially suggested by Head et al. (2010) and recently supported again by Gieg et al. (2014) where several species could be involved.

Although anaerobic benzene biodegradation has been clearly demonstrated in various conditions, the genetic pathways involved are still unclear, despite the finding of a putative gene cluster (Abu Laban et al., 2010; Vogt et al., 2011). So far, no genetic probe targeting functional genes exists to show direct evidence of anaerobic benzene biodegradation. Therefore, field applications of compound-specific isotope analysis (CSIA) and comparison with literature data from well-known biodegradation cases are currently our most powerful diagnostic tools. Significant outputs of this work would concern petroleum companies not only in the context of bioremediation of deep confined aquifers (Declercq et al., 2012) but also for understanding microbial-induced souring in the oil and gas producing reservoirs (Sun et al., 2005; Wilkes et al., 2008; Gieg et al., 2011).

## Conclusion

In this study, it was shown for the first time that a bacterial community composed of only two *Desulfotomaculum* populations can use toluene, ethylbenzene and benzene as sole carbon and energy sources in sulfate-reducing conditions. They constitute the simplest model of anaerobic sulfate-reducing hydrocarbon-degrading anaerobes originating from the deep subterranean environments ever described. While many studies have shown *Pelotomaculum* sp., another genus of *Peptococcaceae*, as key-players in BTEX biodegradation, this work highlights the important role of the genus *Desulfotomaculum* as significant indigenous populations of subsurface habitats, but also as an important agent in the anaerobic degradation of hydrocarbons. In the deep subterranean biosphere, complex litho-autotrophic microbial network promotes reactions for hydrogen interspecies electron transfer. We hypothesize selection of microbial populations able to conduct syntrophic oxidation of organic carbon could be a logical adaptive response of originally litho-autotrophic indigenous microbial communities to the presence of recalcitrant hydrocarbons. Yet, at a time when the exploitation of shale gas and oil is quickly increasing globally, it is necessary to know whether these still poorly undescribed deep subterranean environments have the potential of hydrocarbon degradation, and particularly BTEX. Field monitored natural attenuation (MNA) approaches for subterranean environments should be favored by updating the existing database grouping the identified microbial phylotypes and isotopic values in the context of BTEX biodegradation.

## Author Contributions

TA, SB, and J-F L designed, performed experiments, analyzed data and wrote the paper; DD and DD performed isotopic fractionation experiments; SS-L, BS, and JM performed cultural experiments; DP and CB critically reviewed this paper; MM and ARP designed, analyzed data, supervised the project and wrote the paper; TA, SB, and J-FL are co-first authors.

## Conflict of Interest Statement

The authors declare that the research was conducted in the absence of any commercial or financial relationships that could be construed as a potential conflict of interest.

## References

[B1] Abu Laban N. Selesi D. Jobelius C. Meckenstock R. U. (2009). Anaerobic benzene degradation by Gram-positive sulfate-reducing bacteria. *FEMS Microbiol. Ecol.* 68 300–311. 10.1111/j.1574-6941.2009.00672.x 19416354

[B2] Abu Laban N. Selesi D. Rattei T. Tischler P. Meckenstock R. U. (2010). Identification of enzymes involved in anaerobic benzene degradation by a strictly anaerobic iron-reducing enrichment culture. *Environ. Microbiol.* 12 2783–2796. 10.1111/j.1462-2920.2010.02248.x 20545743

[B3] Abu Laban N. Tan B. F. Dao A. Foght J. (2015). Draft genome sequence of uncultivated *Desulfosporosinus* sp. strain Tol-M, obtained by stable isotope probing using [13C6]toluene. *Genome Announc.* 3:e1422 10.1128/genomeA.01422-14 PMC4299902 25593260

[B4] Ahad J. M. E. Sherwood Lollar B. Edwards E. A. Slater G. F. Sleep B. E. (2000). Carbon isotope fractionation during anaerobic biodegradation of toluene: implications for intrinsic bioremediation. *Environ. Sci. Technol.* 34 892–896. 10.1021/es990797y

[B5] Anderson R. T. Lovley D. R. (2000). Anaerobic bioremediation of benzene under sulfate-reducing conditions in a petroleum-contaminated aquifer. *Environ. Sci. Technol.* 34 2261–2266. 10.1021/es991211a

[B6] Anisimova M. Gascuel O. (2006). Approximate likelihood-ratio test for branches: a fast, accurate, and powerful alternative. *Syst. Biol.* 55 539–552. 10.1080/10635150600755453 16785212

[B7] Aüllo T. Ranchou-Peyruse A. Ollivier B. Magot M. (2013). *Desulfotomaculum* spp. and related gram-positive sulfate-reducing bacteria in deep subsurface environments. *Front. Microbiol.* 4:362 10.3389/fmicb.2013.00362 PMC3844878 24348471

[B8] Baito K. Imai S. Matsushita M. Otani M. Sato Y. Kimura H. (2015). Biogas production using anaerobic groundwater containing a subterranean microbial community associated with the accretionary prism. *Microbial Biotechnol.* 8 835–845. 10.1111/1751-7915.12179 PMC4554471 25267392

[B9] Baker B. J. Moser D. P. MacGregor B. J. Fishbain S. Wagner M. Fry N. K. (2003). Related assemblages of sulfate-reducing bacteria associated with ultradeep gold mines of South Africa and deep basalt aquifers of Washington State. *Environ. Microbiol.* 5 267–277. 10.1046/j.1462-2920.2003.00408.x 12662174

[B10] Balk M. Mehboob F. van Gelder A. H. Rijpstra W. I. C. Damsté J. S. S. Stams A. J. (2010). (Per) chlorate reduction by an acetogenic bacterium, *Sporomusa* sp., isolated from an underground gas storage. *Appl. Microbiol. Biotechnol.* 88 595–603. 10.1007/s00253-010-2788-8 20680263 PMC2924991

[B11] Balk M. van Gelder T. Weelink S. A. Stams A. J. (2008). (Per) chlorate reduction by the thermophilic bacterium *Moorella perchloratireducens* sp. nov., isolated from an underground gas storage. *Appl. Environ. Microbiol.* 74 403–409. 10.1128/AEM.01743-07 17981952 PMC2223267

[B12] Ball H. A. Reinhard M. (1996). Monoaromatic hydrocarbon transformation under anaerobic conditions at Seal Beach, California: laboratory studies. *Environ. Toxicol. Chem.* 15 114–122. 10.1002/etc.5620150207

[B13] Barker J. P. Patrick G. Major D. (1987). Natural attenuation of aromatic hydrocarbons in a shallow sand aquifer. *Ground Water Monit. Remediat.* 7 64–71. 10.1111/j.1745-6592.1987.tb01063.x

[B14] Basso O. Lascourrèges J.-F. Jarry M. Magot M. (2005). The effect of cleaning and disinfecting the sampling well on the microbial communities of deep subsurface water samples. *Environ. Microbiol.* 7 13–21. 10.1111/j.1462-2920.2004.00660.x 15643931

[B15] Basso O. Lascourrèges J.-F. Le Borgne F. Le Goff C. Magot M. (2009). Characterization by culture and molecular analysis of the microbial diversity of a deep subsurface gas storage aquifer. *Res. Microbiol.* 160 107–116. 10.1016/j.resmic.2008.10.010 19056488

[B16] Bergmann F. D. Abu Laban N. M. Meyer A. H. Elsner M. Meckenstock R. U. (2011). Dual (C, H) isotope fractionation in anaerobic low molecular weight (poly) aromatic hydrocarbon (PAH) degradation: potential for field studies and mechanistic implications. *Environ. Sci. Technol.* 45 6947–6953. 10.1021/es201096j 21711028

[B17] Berlendis S. Lascourrèges J.-F. Schraauwers B. Sivadon P. Magot M. (2010). Anaerobic biodegradation of BTEX by original bacterial communities from an underground gas storage aquifer. *Environ. Sci. Technol.* 44 3621–3628. 10.1021/es100123b 20380433

[B18] Borden R. C. Gomez C. A. Becker M. T. (1995). Geochemical indicators of intrinsic bioremediation. *Ground Water* 33 180–189. 10.1111/j.1745-6584.1995.tb00272.x

[B19] Braeckevelt M. Fischer A. Kästner M. (2012). Field applicability of Compound-Specific Isotope Analysis (CSIA) for characterization and quantification of in situ contaminant degradation in aquifers. *Appl. Microbiol. Biotechnol.* 94 1401–1421. 10.1007/s00253-012-4077-1 22573267

[B20] Castresana J. (2000). Selection of conserved blocks from multiple alignments for their use in phylogenetic analysis. *Mol. Biol. Evol.* 17 540–552. 10.1093/oxfordjournals.molbev.a026334 10742046

[B21] Chakraborty R. Coates J. D. (2004). Anaerobic degradation of monoaromatic hydrocarbons. *Appl. Microbiol. Biotechnol.* 64 437–446. 10.1007/s00253-003-1526-x 14735323

[B22] Chakraborty R. O’Connor S. M. Chan E. Coates J. D. (2005). Anaerobic degradation of benzene, toluene, ethylbenzene, and xylene compounds by Dechloromonas strain RCB. *Appl. Environ. Microbiol.* 71 8649–8655. 10.1128/AEM.71.9.5427-5432.2005 16332859 PMC1317370

[B23] Coates J. D. Chakraborty R. Lack J. G. O’Connor S. M. Cole K. A. Bender K. S. (2001). Anaerobic benzene oxidation coupled to nitrate reduction in pure culture by two strains of Dechloromonas. *Nature* 411 1039–1043. 10.1038/35082545 11429602

[B24] Cole J. R. Chai B. Marsh T. L. Farris R. J. Wang Q. Kulam S. A. (2003). The Ribosomal Database Project (RDP-II): previewing a new autoaligner that allows regular updates and the new prokaryotic taxonomy. *Nucleic Acids Res.* 31 442–443. 10.1093/nar/gkg039 12520046 PMC165486

[B25] Coplen T. B. Brand W. A. Gehre M. Gröning M. Meijer H. A. Toman B. (2006). New guidelines for δ 13C measurements. *Anal. Chem.* 78 2439–2441. 10.1021/ac052027c 16579631

[B26] Cunningham J. A. Rahme H. Hopkins G. D. Lebron C. Reinhard M. (2001). Enhanced in situ bioremediation of BTEX-contaminated groundwater by combined injection of nitrate and sulfate. *Environ. Sci. Technol.* 35 1663–1670. 10.1021/es001722t 11329718

[B27] Da Silva M. L. B. Alvarez P. J. J. (2007). Assessment of anaerobic benzene degradation potential using 16S rRNA gene-targeted real-time PCR. *Environ. Microbiol.* 9 72–80. 10.1111/j.1462-2920.2005.01116.x 17227413

[B28] Daumas S. Cord-Ruwish R. Garcia J. L. (1988). *Desulfotomaculum geothermicum* sp. nov., a thermophilic, fatty acid-degrading, sulfate-reducing bacterium isolated with H_2_ from geothermal ground water. *Antonie van Leeuwenhoek* 54 165–178. 10.1007/BF00419203 3395110

[B29] Declercq I. Cappuyns V. Duclos Y. (2012). Monitored Natural Attenuation (MNA) of contaminated soils: state of the art in Europe—a critical evaluation. *Sci. Total Environ.* 426 393–405. 10.1016/j.scitotenv.2012.03.040 22513404

[B30] DeLong E. F. (1992). Archaea in costal marine environments. *Proc. Natl. Acad. Sci. U.S.A.* 89 5685–5689. 10.1073/pnas.89.12.5685 1608980 PMC49357

[B31] Detmers J. Strauss H. Schulte U. Bergmann A. Knittel K. Kuever J. (2004). FISH shows that *Desulfotomaculum* spp. are the dominating sulfate-reducing bacteria in a pristine aquifer. *Microbial Ecol.* 47 236–242. 10.1007/s00248-004-9952-6 15085304

[B32] Dolfing J. Larter S. R. Head I. M. (2008). Thermodynamic constraints on methanogenic crude oil biodegradation. *ISME J.* 452 442–452. 10.1038/ismej.2007.111 18079730

[B33] Edgar R. C. (2004). MUSCLE: multiple sequence alignment with high accuracy and high throughput. *Nucleic Acids Res.* 32 1792–1797. 10.1093/nar/gkh340 15034147 PMC390337

[B34] Edwards E. A. Grbić-Galić D. (1992). Complete mineralization of benzene by aquifer microorganisms under strictly anaerobic conditions. *Appl. Environ. Microbiol.* 58 2663–2666. 1514813 10.1128/aem.58.8.2663-2666.1992 PMC195836

[B35] Ehinger S. Seifert J. Kassahun A. Schmalz L. Hoth N. Schlömann M. (2009). Predominance of *Methanolobus* spp., and *Methanoculleus* spp. in the archaeal communities of saline gas field formation fluids. *Geomicrobiology* 26 326–338. 10.1080/01490450902754441

[B36] Eichler B. Pfennig N. (1986). Characterization of a new platelet-forming purple sulfur bacterium, *Amoebobacter pedioformis* sp. nov. *Arch. Microbiol.* 146 295–300. 10.1007/BF00403233

[B37] Elsner M. Zwank L. Hunkeler D. Schwarzenbach R. P. (2005). A new concept linking observable stable isotope fractionation to transformation pathways of organic pollutants. *Environ. Sci. Technol.* 39 6896–6916. 10.1021/es0504587 16201610

[B38] Fischer A. Herklotz I. Herrmann S. Thullner M. Weelink S. A. B. Stams A. J. M. (2008). Combined carbon and hydrogen isotope fractionation investigations for elucidating benzene biodegradation pathways. *Environ. Sci. Technol.* 42 4356–4363. 10.1021/es702468f 18605555

[B39] Fischer A. Theuerkorn K. Stelzer N. Gehre M. Thullner M. Richnow H. H. (2007). Applicability of stable isotope fractionation analysis for the characterization of benzene biodegradation in a BTEX-contaminated aquifer. *Environ. Sci. Technol.* 41 3689–3696. 10.1021/es061514m 17547198

[B40] Gieg L. M. Fowler S. J. Berdugo-Clavijo C. (2014). Syntrophic biodegradation of hydrocarbon contaminants. *Curr. Opin. Biotechnol.* 27 21–29. 10.1016/j.copbio.2013.09.002 24863893

[B41] Gieg L. M. Jack T. R. Foght J. M. (2011). Biological souring and mitigation in oil reservoirs. *Appl. Microbiol. Biotechnol.* 92 263–282. 10.1007/s00253-011-3542-6 21858492

[B42] Gihring T. M. Moser D. P. Lin L. H. Davidson M. Onstott T. C. Morgan L. (2006). The distribution of microbial taxa in the subsurface water of the Kalahari Shield. *South Africa. Geomicrobiol. J.* 23 415–430. 10.1080/01490450600875696

[B43] Greksák M. Šmigáò P. Kozánková J. Buzek F. Onderka V. Wolf I. (1990). “Methanogenic bacteria and their activity in a subsurface reservoir of town gas,” in *Microbiology and Biochemistry of Strict Anaerobes Involved in Interspecies Hydrogen Transfer* eds Bélaich J.-P. Bruschi M. Garcia J.-L. (Berlin: Springer).

[B44] Griebler C. Malard F. Lefébure T. (2014). Current developments in groundwater ecology - from biodiversity to ecosystem function and services. *Curr. Opin. Biotechnol.* 27 159–167. 10.1016/j.copbio.2014.01.018 24590188

[B45] Grobkopf R. Stubner S. Liesack W. (1998). Novel Euryarchaeotal lineages detected on rice roots and in the anoxic bulk soil of flooded rice microcosms. *Appl. Environ. Microbiol.* 64 4983–4989. 9835592 10.1128/aem.64.12.4983-4989.1998 PMC90952

[B46] Guan J. Xia L. P. Wang L. Y. Liu J. F. Gu J. D. Mu B. Z. (2013). Diversity and distribution of sulfate-reducing bacteria in four petroleum reservoirs detected by using 16S rRNA and dsrAB genes. *Int. Biodeterior. Biodegradation* 76 58–66. 10.1016/j.ibiod.2012.06.021

[B47] Guindon S. Gascuel O. (2003). A simple, fast, and accurate algorithm to estimate large phylogenies by maximum likelihood. *Syst. Biol.* 52 696–704. 10.1080/10635150390235520 14530136

[B48] Haouari O. Fardeau M.-L. Cayol J.-L. Casiot C. Elbaz-Poulichet F. Hamdi M. (2008). *Desulfotomaculum hydrothermale* sp. nov., a thermophilic sulfate-reducing bacterium isolated from a terrestrial Tunisian hot spring. *Int. J. Syst. Evol. Microbiol.* 58 2529–2535. 10.1099/ijs.0.65339-0 18984688

[B49] Head I. M. Jones D. M. Larter S. R. (2003). Biological activity in the deep subsurface and the origin of heavy oil. *Nature* 426 344–352. 10.1038/nature02134 14628064

[B50] Head I. M. Larter S. R. Gray N. D. Sherry A. Adams J. J. Aitken C. M. (2010). “Hydrocarbon degradation in petroleum reservoirs,” in *Handbook of Hydrocarbon and Lipid Microbiology* ed. Timmis K. N. (Berlin: Springer) 3097–3109.

[B51] Heider J. Spormann A. M. Beller H. R. Widdel F. (1999). Anaerobic bacterial metabolism of hydrocarbons. *FEMS Microbiol. Rev.* 22 459–473. 10.1111/j.1574-6976.1998.tb00381.x

[B52] Hermann S. Kleinsteuber S. Neu T. R. Richnow H. H. Vogt C. (2008). Enrichment of anaerobic benzene-degrading microorganisms by in situ microcosms. *FEMS Microbiol. Ecol.* 63 94–106. 10.1111/j.1574-6941.2007.00401.x 18081593

[B53] Hoehler T. M. Jørgensen B. B. (2013). Microbial life under extreme energy limitation. *Nat. Rev. Microbiol.* 11 83–94. 10.1038/nrmicro2939 23321532

[B54] Holmes D. E. Risso C. Smith J. A. Lovley D. R. (2011). Anaerobic oxidation of benzene by the hyperthermophilic archaeon *Ferroglobus placidus*. *Appl. Environ. Microbiol.* 77 5926–5933. 10.1128/AEM.05452-11 21742914 PMC3165377

[B55] Hubbard C. G. Cheng Y. Engelbrekston A. Druhan J. L. Li L. Ajo-Franklin J. B. (2014). Isotopic insights into microbial sulfur cycling in oil reservoirs. *Front. Microbiol.* 5:480 10.3389/fmicb.2014.00480 PMC4168720 25285094

[B56] Hunkeler D. Andersen N. Aravena R. Bernasconi S. M. Butler B. J. (2001). Hydrogen and carbon isotope fractionation during aerobic biodegradation of benzene. *Environ. Sci. Technol.* 35 3462–3467. 10.1021/es0105111 11563647

[B57] Imfeld G. Kopinke F. D. Fischer A. Richnow H. H. (2014). Carbon and hydrogen isotope fractionation of benzene and toluene during hydrophobic sorption in multistep batch experiments. *Chemosphere* 107 454–461. 10.1016/j.chemosphere.2014.01.063 24726480

[B58] Ivanova A. E. Borzenkov I. A. Tarasov A. L. Milekhina E. I. Belyaev S. S. (2007). A microbiological study of an underground gas storage in the process of gas extraction. *Microbiology* 76 461–468. 10.1134/S002626170704011X 17974210

[B59] Jones D. M. Head I. M. Gray N. D. Adams J. J. Rowan A. K. Aitken C. M. (2008). Crude-oil biodegradation via methanogenesis in subsurface petroleum reservoirs. *Nature* 451 176–180. 10.1038/nature06484 18075503

[B60] Kasai Y. Kodama Y. Takahata Y. Hoaki T. Watanabe K. (2007). Degradative capacities and bioaugmentation potential of an anaerobic benzene-degrading bacterium strain DN11. *Environ. Sci. Technol.* 41 6222–6227. 10.1021/es062842p 17937306

[B61] Kelley C. A. Hammer B. T. Coffin R. B. (1997). Concentrations and stable isotope values of BTEX in gasoline-contaminated groundwater. *Environ. Sci. Technol.* 31 2469–2472. 10.1021/es960635r

[B62] Kleinsteuber S. Schleinitz K. M. Breitfeld J. Harms H. Richnow H. H. Vogt C. (2008). Molecular characterization of bacterial communities mineralizing benzene under sulphate-reducing conditions. *FEMS Microbiol. Ecol.* 66 143–157. 10.1111/j.1574-6941.2008.00536.x 18637040

[B63] Kniemeyer O. Fisher T. Wilkes H. Glöckner F. O. Widdel F. (2003). Anaerobic degradation of ethylbenzene by a new type of marine sulfate-reducing bacterium. *Appl. Environ. Microbiol.* 69 760–768. 10.1128/AEM.69.2.760-768.2003 12570993 PMC143655

[B64] Kunapuli U. Lueders T. Meckenstock R. U. (2007). The use of stable istope probing to identify key iron-reducing microorganisms involved in anerobic benzene degradtion. *ISME J.* 1 643–653. 10.1038/ismej.2007.73 18043671

[B65] Kuppardt A. Kleinsteuber S. Vogt C. Lüders T. Harms H. Chatzinotas A. (2014). Phylogenetic and functional diversity within toluene-degrading, sulphate-reducing consortia enriched from a contaminated aquifer. *Microbiol. Ecol.* 68 222–234. 10.1007/s00248-014-0403-8 24623528

[B66] Lane D. J. (1991). “16S/23S rRNA sequencing,” in *Nucleic Acid Techniques in Bacterial Systematics* eds Stackebrandt E. Goodfellow M. (Chichester: John Wiley) 115–175.

[B67] Liu A. Garcia-Dominguez E. Rhine E. Young L. (2004). A novel arsenate respiring isolate that can utilize aromatic substrates. *FEMS Microbiol. Ecol.* 48 323–332. 10.1016/j.femsec.2004.02.008 19712302

[B68] Liu Y. Karnauchow T. M. Jarrell K. F. Balkwill D. L. Drake G. R. Ringelberg D. (1997). Description of two new thermophilic *Desulfotomaculum* spp., *Desulfotomaculum putei* sp. nov., from a deep terrestrial subsurface, and *Desulfotomaculum luciae* sp. nov., from a hot spring. *Int. J. Syst. Bacteriol.* 47 615–621. 10.1099/00207713-47-3-615

[B69] Magot M. Ollivier B. Patel B. K. C. (2000). Microbiology of petroleum reservoirs. *Antonie Van Leeuwenhoek* 77 103–116. 10.1023/A:1002434330514 10768470

[B70] Mancini S. A. Devine C. E. Elsner M. Nandi M. E. Ulrich A. C. Edwards E. A. (2008). Isotopic evidence suggests different initial reaction mechanisms for anaerobic benzene biodegradation. *Environ. Sci. Technol.* 42 8290–8296. 10.1021/es801107g 19068808

[B71] Mancini S. A. Ulrich A. C. Lacrampe-Couloume G. Sleep B. Edwards E. A. Sherwood Lollar B. (2003). Carbon and hydrogen isotopic fractionation during anaerobic biodegradation of benzene. *Appl. Environ. Microbiol.* 69 191–198. 10.1128/AEM.69.1.191-198.2003 12513995 PMC152413

[B72] Mayumi D. Mochimaru H. Yoshioka H. Sakata S. Maeda H. Miyagawa Y. (2011). Evidence for syntrophic acetate oxidation coupled to hydrogenotrophic methanogenesis in the high-temperature petroleum reservoir of Yabase oil field (Japan). *Environ. Microbiol.* 13 1995–2006. 10.1111/j.1462-2920.2010.02338.x 20860731

[B73] McMahon S. Parnell J. (2014). Weighing the deep continental biosphere. *FEMS Microbiol. Ecol.* 87 113–120. 10.1111/1574-6941.12196 23991863

[B74] Meckenstock R. U. (1999). Fermentative toluene degradation in anaerobic defined syntrophic cocultures. *FEMS Microbiol. Lett.* 177 67–73. 10.1111/j.1574-6968.1999.tb13715.x 10436924

[B75] Meckenstock R. U. Morasch B. Griebler C. Richnow H. H. (2004). Stable isotope fractionation analysis as a tool to monitor biodegradation in contaminated acquifers. *J. Contam. Hydrol.* 75 215–255. 10.1016/j.jconhyd.2004.06.003 15610901

[B76] Morasch B. Schink B. Tebbe C. C. Meckenstock R. U. (2004). Degradation of o-xylene and m-xylene by a novel sulfate-reducer belonging to the genus *Desulfotomaculum*. *Arch. Microbiol.* 181 407–417. 10.1007/s00203-004-0672-6 15127183

[B77] Moser D. P. Gihring T. M. Brockman F. J. Fredrickson J. K. Balkwill D. L. Dollhopf M. E. (2005). *Desulfotomaculum* and *Methanobacterium* spp. dominate a 4- to 5-kilometer-deep fault. *Appl. Environ. Microbiol.* 71 8773–8783. 10.1128/AEM.71.12.8773-8783.2005 16332873 PMC1317344

[B78] Moser D. P. Onstott T. C. Fredrickson J. K. Brockman F. J. Balkwill D. L. Drake G. R. (2003). Temporal shifts in the geochemistry and microbial community structure of an ultradeep mine borehole following isolation. *Geomicrobiol. J.* 20 517–548. 10.1080/713851170

[B79] Musat F. Widdel F. (2008). Anaerobic degradation of benzene by a marine sulfate-reducing enrichment culture, and cell hybridization of the dominant phylotype. *Environ. Microbiol.* 10 10–19. 10.1111/j.1462-2920.2007.01425.x 18211263

[B80] Nazina T. N. Shestakova N. M. Grigor’yan A. A. Mikhailova E. M. Tourova T. P. Poltaraus A. B. (2006). Phylogenetic diversity and activity of anaerobic microorganisms of high-temperature horizons of the Dagang oil field (PR China). *Microbiology* 75 55–65. 10.1134/S0026261706010115 16579447

[B81] Nilsen R. K. Torsvik T. Lien T. (1996). *Desulfotomaculum thermocisternum* sp. nov., a sulfate-reducer isolated from a hot North Sea oil reservoir. *Int. J. Syt. Bacteriol.* 46 397–402. 10.1099/00207713-46-2-397

[B82] Ogg C. D. Patel B. K. C. (2011). *Desulfotomaculum varum* sp. nov., a moderately thermophilic sulfate-reducing bacterium isolated from a microbial mat colonizing a Great Artesian Basin bore well runoff channel. *Biotechnology* 1 139–149. 10.1007/s13205-011-0017-5 PMC3339622 22611525

[B83] Oka A. R. Phelps C. D. McGuinness L. M. Mumford A. Young L. Y. Kerkhof L. J. (2008). Identification of critical members in a sulfidogenic benzene-degrading consortium by DNA stable isotope probing. *Appl. Environ. Microbiol.* 74 6476–6480. 10.1128/AEM.01082-08 18757571 PMC2570285

[B84] Onstott T. C. Hinton S. M. Silver B. J. Kingjr H. E. (2010). Coupling hydrocarbon degradation to anaerobic respiration and mineral diagenesis: theoretical constraints. *Geobiology* 8 69–88. 10.1111/j.1472-4669.2009.00224.x 20055900

[B85] O’Sullivan L. A. Roussel E. G. Weightman A. J. Webster G. Hubert C. R. J. Bell E. (2015). Survival of *Desulfotomaculum* spores from estuarine sediments after serial autoclaving and high-temperature exposure. *ISME J.* 9 922–933. 10.1038/ismej.2014.190 25325382 PMC4817712

[B86] Pedersen K. (2000). Exploration of deep intraterrestrial microbial life: current perspectives. *FEMS Microbiol. Lett.* 185 9–16. 10.1111/j.1574-6968.2000.tb09033.x 10731600

[B87] Pfennig N. Widdel F. Trüper H. (1981). “The dissimilatory sulfate-reducing bacteria,” in *The Prokaryotes* eds Starr M. P. Stolp H. Türper H. G. Balows A. Schlegel H. G. (Berlin: Springer) 926–940.

[B88] Phelps C. D. Kerkhof L. J. Young L. Y. (1998). Molecular characterization of a sulfate-reducing consortium which mineralizes benzene. *FEMS Microbiol. Ecol.* 27 269–279. 10.1111/j.1574-6941.1998.tb00543.x

[B89] Rabus R. Heider J. (1998). Initial reactions of anaerobic metabolism of alkylbenzenes in denitrifying and sulfate-reducing bacteria. *Arch. Microbiol.* 170 377–384. 10.1007/s002030050656

[B90] Robertson W. J. Franzmann P. D. Mee B. J. (2000). Spore-forming Desulfosporosinus-like sulfate-reducing bacteria from a shallow aquifer contaminated with gasoline. *J. Appl. Bacteriol.* 88 248–259. 10.1046/j.1365-2672.2000.00957.x 10735993

[B91] Ruiz-Aguilar G. M. L. O’Reilly K. Alvarez P. J. J. (2003). A comparison of benzene and toluene plume lengths for sites contaminated with regular vs. ethanol-amended gasoline. *Ground Water Monit. Remediat.* 23 48–53. 10.1111/j.1745-6592.2003.tb00782.x

[B92] Salinero K. K. Keller K. Feil W. S. Feil H. Trong S. Di Bartolo G. (2009). Metabolic analysis of the soil microbe *Dechloromonas aromatic* str. RCB: indications of a surprisingly complex life-style and cryptic anaerobic pathways for aromatic degradation. *BMC Genomics* 10:351 10.1186/1471-2164-10-351 PMC2907700 19650930

[B93] Scow K. M. Hicks K. A. (2005). Natural attenuation and enhanced bioremediation of organic contaminants in groundwater. *Curr. Opin. Biotechnol.* 16 246–253. 10.1016/j.copbio.2005.03.009 15961025

[B94] Selesi D. Meckenstock R. U. (2009). Anaerobic degradation of the aromatic hydrocarbon biphenyl by a sulfate-reducing enrichment culture. *FEMS Microbiol. Ecol.* 68 86–93. 10.1111/j.1574-6941.2009.00652.x 19187215

[B95] Shen H. Sewell G. W. (2005). Reductive biotransformation of tetrachloroethene to ethene during anaerobic degradation of toluene: experimental evidence and kinetics. *Environ. Sci. Technol.* 39 9286–9294. 10.1021/es050390v 16382954

[B96] Stackebrandt E. Goebel B. M. (1994). Taxonomic note: a place for DNA-DNA reassociation and 16S rRNA sequence analysis in the present species definition in bacteriology. *Int. J. Syst. Bacteriol.* 44 846–849. 10.1099/00207713-44-4-846

[B97] Stevens T. O. McKinley J. P. (1995). Lithoautotrophic microbial ecosystems in deep basalt aquifers. *Science* 270 450–455.

[B98] Sun W. Sun X. Cupples A. M. (2013). Presence, diversity and enumeration of functional genes (bssA and bamA) relating to toluene degradation across a range of redox conditions and inoculum sources. *Biodegradation* 25 189–203. 10.1007/s10532-013-9651-4 23728713

[B99] Sun Y. Chen Z. Xu S. Cai P. (2005). Stable carbon and hydrogen isotopic fractionation of individual n-alkanes accompanying biodegradation: evidence from a group of progressively biodegraded oils. *Organ. Geochem.* 36 225–238. 10.1016/j.orggeochem.2004.09.002

[B100] Taubert M. Vogt C. Wubet T. Kleinsteuber S. Tarkka M. T. Harms H. (2012). Protein-SIP enables time-resolved analysis of the carbon flux in a sulfate-reducing, benzene-degrading microbial consortium. *ISME J.* 6 2291–2301. 10.1038/ismej.2012.68 22791237 PMC3504967

[B101] van der Zaan B. M. Talarico Saia F. Stams A. J. M. Plugge C. M. de Vos W. M. Smidt H. (2012). Anaerobic benzene degradation under denitrifying conditions: peptococcaceae as dominant benzene degraders and evidence for a syntrophic process. *Environ. Microbiol.* 14 1171–1181. 10.1111/j.1462-2920.2012.02697.x 22296107

[B102] Vetriani C. Jannasch H. W. Macgregor B. J. Stahl D. A. Reysenbach A. L. (1999). Population structure and phylogenetic characterization of marine benthic Archaea in deep-sea sediments. *Appl. Environ. Microbiol.* 65 4375–4384. 10508063 10.1128/aem.65.10.4375-4384.1999 PMC91581

[B103] Vogt C. Kleinsteuber S. Richnow H. H. (2011). Anaerobic benzene degradation by bacteria. *Microbial Biotechnol.* 4 710–724. 10.1111/j.1751-7915.2011.00260.x PMC3815408 21450012

[B104] von Netzer F. Pilloni G. Kleindienst S. Krüger M. Knittel K. Gründger F. (2013). Enhanced gene detection assays for Fumarate-Adding Enzymes allow uncovering of anaerobic hydrocarbon degraders in terrestrial and marine systems. *Appl. Environ. Microbiol.* 79 543–552. 10.1128/AEM.02362-12 23124238 PMC3553772

[B105] Warren E. Bekins B. Godsy E. Smith V. (2004). Inhibition of acetoclastic methanogenesis in crude oil- and creosote-contaminated groundwater. *Bioremediation J.* 8 1–11. 10.1080/10889860490465840

[B106] Weelink S. A. B. van Eekert M. H. A. Stams A. J. M. (2010). Degradation of BTEX by anaerobic bacteria: physiology and application. *Rev. Environ. Sci. Biotechnol.* 9 359–385. 10.1007/s11157-010-9219-2

[B107] Weisburg W. G. Barns S. M. Pelletier D. A. Lane D. J. (1991). 16S ribosomal DNA amplification for phylogenetic study. *J. Bacteriol.* 173 697–703. 1987160 10.1128/jb.173.2.697-703.1991 PMC207061

[B108] Whitman W. B. Coleman D. C. Wiebe W. J. (1998). Prokaryotes: the unseen majority. *Proc. Natl. Acad. Sci. U.S.A.* 95 6578–6583. 10.1073/pnas.95.12.6578 9618454 PMC33863

[B109] Widdel F. (2006). “The genus *Desulfotomaculum*,” in *The Prokaryotes* Vol. 4 eds DeLong E. F. Lory S. Stackebrandt E. Thompson F. (New York, NY: Springer) 787–794.

[B110] Widdel F. Knittel K. Galushko A. (2010). “Anaerobic hydrocarbon-degrading microorganisms: an overview,” in *Handbook of Hydrocarbon and Lipid Microbiology* eds Timmis K. N. McGenity T. J. van der Meer J. R. de Lorenzo V. (Berlin: Springer) 1997–2021.

[B111] Widdel F. Rabus R. (2001). Anaerobic biodegradation of saturated and aromatic hydrocarbons. *Curr. Opin. Biotechnol.* 12 259–276. 10.1016/S0958-1669(00)00209-3 11404104

[B112] Wilkes H. Vieth A. Elias R. (2008). Constraints on the quantitative assessment of in-reservoir biodegradation using compound-specific stable carbon isotopes. *Organ. Geochem.* 39 1215–1221. 10.1016/j.orggeochem.2008.02.013

[B113] Wilkins M. J. Daly R. A. Mouser P. J. Trexler R. Sharma S. Cole D. R. (2014). Trends and future challenges in sampling the deep terrestrial biosphere. *Front. Microbiol.* 5:481 10.3389/fmicb.2014.00481 PMC4162470 25309520

[B114] Winderl C. Penning H. von Netzer F. Meckenstock R. U. Lueders T. (2010). DNA-SIP identities sulphate-reducing Clostridia as important toluene degraders in tar-oil-contaminated aquifer sediment. *ISME J.* 4 1314–1325. 10.1038/ismej.2010.54 20428224

[B115] Winderl C. Schaefer S. Lueders T. (2007). Detection of anaerobic toluene and hydrocarbon degraders in contaminated aquifers using benzylsuccinate synthase (bssA) genes as a functional marker. *Environ. Microbiol.* 9 1035–1046. 10.1111/j.1462-2920.2006.01230.x 17359274

[B116] Zhang T. Bain T. S. Nevin K. P. Barlett M. A. Lovley D. R. (2012). Anaerobic benzene oxidation by Geobacter species. *Appl. Environ. Microbiol.* 78 8304–8310. 10.1128/AEM.02469-12 23001648 PMC3497359

[B117] Zwank L. Berg M. Elsner M. Schmidt T. C. Schwarzenbach R. P. Haderlein S. B. (2004). New evaluation scheme for two-dimensional isotope analysis to decipher biodegradation processes: application to groundwater contamination by MTBE. *Environ. Sci. Technol.* 39 1018–1029. 10.1021/es049650j 15773473

